# Abnormal voxel-mirrored homotopic connectivity in first-episode major depressive disorder using fMRI: a machine learning approach

**DOI:** 10.3389/fpsyt.2023.1241670

**Published:** 2023-09-12

**Authors:** Qing Chen, Yanmeng Bi, Weixin Yan, Shuhui Wu, Ting Xia, Yuhua Wang, Sha Huang, Chuying Zhou, Shuwen Xie, Shanshan Kuang, Wen Kong, Zhiping Lv

**Affiliations:** ^1^School of Traditional Chinese Medicine, Southern Medical University, Guangzhou, China; ^2^College of Integrated Traditional Chinese and Western Medicine, Jining Medical University, Jining, China; ^3^The First Affiliated Hospital of Guangzhou University of Chinese Medicine, Guangzhou, China; ^4^The Second Affiliated Hospital of Hunan University of Chinese Medicine, Changsha, China; ^5^Guangzhou Hospital of Integrated Chinese and Western Medicine, Guangzhou, China

**Keywords:** major depressive disorder, voxel-mirrored homotopic connectivity, support vector machine, functional magnetic resonance imaging, resting-state

## Abstract

**Objective:**

To explore the interhemispheric information synergy ability of the brain in major depressive disorder (MDD) patients by applying the voxel-mirrored homotopic connectivity (VMHC) method and further explore the potential clinical diagnostic value of VMHC metric by a machine learning approach.

**Methods:**

52 healthy controls and 48 first-episode MDD patients were recruited in the study. We performed neuropsychological tests and resting-state fMRI scanning on all subjects. The VMHC values of the symmetrical interhemispheric voxels in the whole brain were calculated. The VMHC alterations were compared between two groups, and the relationship between VMHC values and clinical variables was analyzed. Then, abnormal brain regions were selected as features to conduct the classification model by using the support vector machine (SVM) approach.

**Results:**

Compared to the healthy controls, MDD patients exhibited decreased VMHC values in the bilateral middle frontal gyrus, fusiform gyrus, medial superior frontal gyrus and precentral gyrus. Furthermore, the VMHC value of the bilateral fusiform gyrus was positively correlated with the total Hamilton Depression Scale (HAMD). Moreover, SVM analysis displayed that a combination of all clusters demonstrated the highest area under the curve (AUC) of 0.87 with accuracy, sensitivity, and specificity values of 86.17%, 76.74%, and 94.12%, respectively.

**Conclusion:**

MDD patients had reduced functional connectivity in the bilateral middle frontal gyrus, fusiform gyrus, medial superior frontal gyrus and precentral gyrus, which may be related to depressive symptoms. The abnormality in these brain regions could represent potential imaging markers to distinguish MDD patients from healthy controls.

## 1. Introduction

As a common and debilitating mental disease, major depressive disorder (MDD) is characterized by persistently depressed mood, lack of interest, low energy, and cognitive impairment (1). It has high rates of occurrence, impairment and recurrence. Currently, there are more than around 350 million MDD patients worldwide, and the number of patients is still increasing annually (2). According to the World Health Organization, it is estimated that MDD can reach the first incidence rate among mental disorders in the world by 2030, which will seriously threaten economic development and social stability (3, 4). Previous study showed that MDD was a systematic disease involving in multiple neural circuits, which may be related to genetic factors, environmental factors, psychological factors, and abnormal nerve development (5). Although many studies have been performed on the genetics, neurobiochemistry and neuroendocrinology of MDD (6–10), the pathogenesis is still unclear. The diagnosis of MDD is mainly based on the subjective feelings of patients and the evaluation of depression scales depending on the experience of clinicians. Hence, it is an urgent problem to explore the pathogenesis of MDD and find appropriate objective diagnostic markers.

The traditional imaging indicators of structural magnetic resonance imaging (MRI) are insufficient as markers for MDD diagnosis due to the lack of organic lesions. In recent years, resting-state functional magnetic resonance imaging (rs-fMRI) has developed rapidly, providing new ideas. Rs-fMRI is a non-invasive brain imaging technology reflecting the brain functional activity by measuring the hemodynamic and metabolic changes based on blood oxygen level-dependent (11). It has good repeatability and very high spatial resolution. Additionally, subjects do not need to perform specific tasks during the scanning progress. This technology can explore the pathogenesis of diseases from the perspective of neuroimaging and provide an effective means to find neuroimaging markers. Thus, it has been widely used in the research of neuropsychiatric diseases, such as bipolar disorder (12, 13), autism (14, 15), and Alzheimer’s disease (16, 17). It can also be a particularly useful tool for investigating differences between MDD patients and healthy controls (HCs). Previous studies on MDD have revealed that there were structural and functional changes in many brain regions, mainly involving the altered prefrontal cortex, amygdala, hippocampus, corpus striatum, and other brain regions (18–22).

To date, common traditional imaging data analysis methods include amplitude of low-frequency fluctuation (ALFF), regional homogeneity (ReHo), degree centrality (DC) and so on. The methods are mainly utilized to observe brain functional changes in MDD patients from a local perspective. But voxel-mirror homotopic connectivity (VMHC) is a reliable and reproducible measurement from the whole brain level which has been developed rapidly recently (23). It has been applied for neuropsychiatric diseases, such as anxiety disorder (24), autism (25), addiction (26), obsessive-compulsive disorder (27, 28), and Schizophrenia (29). Through the method, the functional connections can be quantified between each voxel in the one hemisphere and the mirror voxel in the other hemisphere at resting state and the intensity reflects the synergy between the hemispheres. In other word, it mainly reflects the information exchange and coordination function between hemispheres by describing the high synchronization of spontaneous activities in the symmetrical regions of the left and right hemispheres. The good coordination of brain regions between hemispheres plays an important role in integrating cognitive and behavioral related brain functions. Therefore, the study of homotopic functional connection across the cerebral hemispheres might help to further understand the neural mechanisms of MDD.

As a supervised machine learning algorithm, support vector machine (SVM) has unique advantages in dealing with small-sample, high-dimensional, and nonlinear data problems for classification (30). It can determine the optimal segmentation hyperplane in the feature space of data samples to maximize the distance between the hyperplane and various types of samples based on the statistical learning theory and the principle of structural risk minimization. Compared to traditional statistical analysis techniques, it has a simple structure, optimal global solution and high generalization ability as a multivariate pattern analysis approach. Furthermore, it enables programs to learn from data sets and perform tasks without direct users input, which has been applied in the discriminant analysis of various neuropsychiatric diseases (31). As we all know, several studies have reported that VMHC method was applied for the different types of MDD (32–37). However, our study is the first to combine VMHC metric and SVM method to evaluate the classification ability in the first-episode MDD patients without prior assumptions.

In the present study, we aimed to explore the possible neuroimaging mechanism of MDD and identify whether the altered brain regions could be used to discriminate between the first-episode MDD patients and HCs. Firstly, the VMHC approach was applied to identify the functional connectivity between the hemispheres. Next, we used correlation analyses to reveal the relationship between abnormal homotopic connectivity and clinical characteristics. Finally, we discussed the VMHC value in altered brain regions as potential neuroimaging markers by the SVM method. The study will deepen our understanding of neural mechanism changes in MDD.

## 2. Methods

### 2.1. Participants

We recruited 48 first-episode MDD patients aged 18–55 years from the traditional Chinese medicine clinic and psychiatric department of Guangdong Sanjiu Brain Hospital, and 52 healthy volunteers from the community through advertisement. This study lasted from May 2017 to August 2018. Before the screening, all subjects signed a written statement of informed consent. This study received ethical approvals from the Ethics Committee of Guangdong Sanjiu Brain Hospital and the Ethics Committee of Southern Medical University. And it was registered on the Chinese clinical trial website (http://www.chictr.org.cn, registration number: ChiCTR-IPR-14005427).

All participants included in this study were right-handed. The Diagnostic and Statistical Manual of Mental Disorders, Fourth Edition-Text Revision (DSM-IV-TR) was used to make the diagnoses of the first-episode MDD patients. The participants met the following inclusion criteria: (1) HAMD score of >20; (2) the course of disease of >2 weeks; (3) no psychiatric drugs intake; (4) no neurological or other psychiatric disorders and history of substance dependence; (5) no organic brain diseases; (6) no history of manic or hypomanic episodes; (7) no history of psychiatric illness among their first-degree relatives; and (8) no MRI contraindications, such as electronic implants, various metals or claustrophobia. Pregnant and lactating women were also excluded.

The HCs met the following inclusion criteria: (1) a comprehensive physical examination conducted before the experiment with all examination results being normal; (2) the total score of HAMD of <7; (3) no mood disorders or neurological disorders; (4) no family history of psychiatric illness among their first-degree relatives; (5) no drugs intake 2 weeks before the experiment; and (6) no MRI contraindications. Furthermore, pregnant and lactating women were excluded. All subjects completed 24 items of the Hamilton Depression Scale (HAMD-24) and Self-Rating Depression Scale (SDS).

### 2.2. MRI data acquisition

In this study, the MRI imaging data was collected using a GE 3 T Signa HDXT superconducting magnetic resonance scanner. During the scanning process, all subjects were instructed to stay awake, lie flat and close their eyes without thinking as much as possible. Their heads were fixed with sponge pads to reduce head movement and equipped with sound insulation earplugs. The sagittal 3D-BRAVO sequence was used for brain 3D-T1WI scanning. The scanning parameters of structural phase were as follows: repetition time (TR) = 8.8 ms, time to Echo (TE) = 3.5 ms, field of view (FOV) = 256 mm × 256 mm, voxel size = 1 mm × 1 mm × 1 mm, flip angle = 13°, matrix = 256 × 256, slices number = 184. Gradient echo planar imaging pulse sequence was used to obtain rs-fMRI imaging data. The front and rear joint lines were taken as the scanning baseline, and oblique axial scanning was performed. The scanning range was from the parietal cranium to the foramen magnum of the subjects. The scanning took about eight minutes and the scanning parameters of functional phase were as follows: TR/TE = 2000 ms/30 ms, FOV = 24 cm × 24 cm, flip angle = 90°, slices number = 33, slice thickness = 5 mm, gap = 0.6 mm, matrix = 64 × 64, time points = 240.

### 2.3. Data preprocessing

RESTplus v1.25 (38) and SPM12 software^1^ were used for data preprocessing based on MATLAB R2017b platform. The preprocessing steps included: (1) converting DICOM format data to NIFTI format data; (2) removing the first 10 time points to minimize the impact from the initial signal volatility; (3) taking the middle slice as a reference for slice timing; (4) realigning for head movement correction; (5) spatial normalizing by using an echo planner imaging (EPI) template developed by the Canadian Montreal Neuroscience Institute; (6) smoothing by using the 6 × 6 × 6 mm^3^ Gaussian smoothing kernel for improving the signal to noise ratio of data; (7) detrending; (8) nuisance covariates regression including the head movement by using Friston 24 parameter (39); and (9) bandpass filtering to reduce the impact of low-frequency drift and high-frequency noise (0.01–0.08 Hz). One HC and five MDD patients were excluded due to excessive head movements that the translation was >2.5 mm, or rotation was >2.5° in each direction during the scanning process. Consequently, 43 patients and 51 HCs were included in the statistical analyses.

### 2.4. VMHC calculation

VMHC analysis was performed based on the DPARSF 6.2 software (40). Firstly, the time series of each voxel were extracted in the one hemisphere of the participants. And then, Pearson correlation coefficient was calculated between the time series and the corresponding time series in the symmetric hemisphere. Subsequently, the obtained correlation coefficient was converted to a *Z* value through Fisher Z transformation in order to generate the VMHC map of the entire brain for each participant. Finally, the average VMHC value of each participant can be extracted for group comparison.

### 2.5. Statistical analysis

Clinical and neuroimaging data were compared between MDD patients and HCs. SPSS 25.0 software (Chicago, IL) was used to analyze the clinical data of the participants. We analyzed neuroimaging data utilizing RESTplus v1.25 software on MATLAB r2017b platform. The continuous data according with the normal distribution and homogeneity of variance were analyzed by two independent sample t-test, and the categorical data was analyzed by χ2 test. The mean and standard deviation were expressed for continuous data. Whereas the median and interquartile range were expressed for counting data. We used the gender, age, and education of subjects as covariates for rs-fMRI data if the two groups differed statistically from one another. The test results were corrected by Gaussian random field (GRF) multiple comparison correction. We considered that voxel *p* of <0.005 and cluster *p* of <0.05 were statistically significant. The VMHC values of abnormal brain regions were extracted for further correlation analysis and classification.

As a supervised machine learning, the SVM method is a common way to explore the best boundaries between two categories and to solve binary classification problems. The method was applied to test whether extracted VMHC could discriminate between MDD patients and healthy controls. The categorization procedure included training and testing. First, abnormal VMHC were utilized as features to establish the hyperplane and the radial basis function (RBF) kernel was applied in the SVM model. The best parameters for the training dataset, including c (penalty coefficient) and g (gamma), were chosen by the grid search approach. Second, an optimal hyperplane which developed from the training data was applied to a new testing dataset in order to assess the performance of the classification. We used a “leave-one-out” method to produce results with the best levels of accuracy, sensitivity and specificity by the LIBSVM software package in MATLAB r2017b platform. The predictive performance of the SVM model was shown by the area under the receiver operating curve (AUC).

## 3. Results

### 3.1. Clinical characteristics

Demographic and clinical characteristics between the two groups were presented in Table 1. The age and sex composition ratios did not significantly differ between the two groups (*p* > 0.05), but there were significant differences in the education level, HAMD-24, and SDS (*p* < 0.05).

**Table 1:** Demographics and clinical characteristics of all subjects.

Group	Age (years)	Gender (male/female)	Education (years)	Duration (weeks)	HAMD	SDS
HCs	28.92 ± 7.12	22/29	13.02 ± 2.96	NA	2.53 ± 1.71	25.65 ± 3.70
MDD	31.12 ± 10.35	20/23	11.53 ± 2.50	33.95 ± 41.60	28.14 ± 3.03	75.26 ± 3.78
t/χ^2^	−1.176	0.107	2.635	NA	−49.214	−64.094
*p* value	0.243	0.743	0.01	NA	< 0.0001	< 0.0001

Data are presented as Mean ± SD; N/A, not applicate.

### 3.2. VMHC comparison

**Figure 1:**
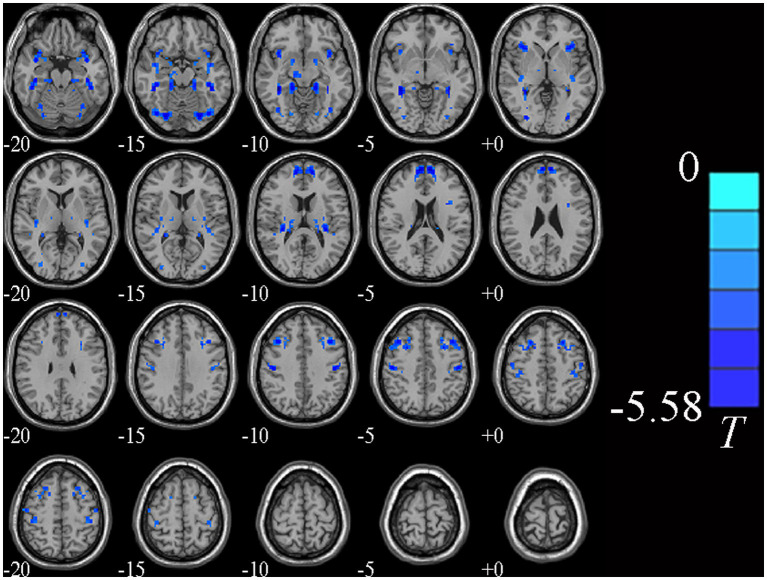
Brain regions showing significantly different VMHC values between two groups. Cold colors indicate decreased VMHC values (voxel *p* < 0.005, cluster *p* < 0.05, GRF correction, cluster size >64 voxels).

Individual whole-brain VMHC values of MDD patients were compared between MDD patients and HCs. Additionally, we took the education level as a covariate in the statistical analysis of rs-fMRI data. MDD patients had lower VMHC values in the bilateral middle frontal gyrus (MFG), fusiform gyrus (FG), medial superior frontal gyrus (MSFG) and precentral gyrus (PG) (GRF correction, voxel *p* < 0.005, cluster *p* < 0.05, cluster size >64) relative to HCs, as shown in Table 2 and Figure 1.

**Table 2:** Abnormal brain regions in the MDD patients compared to HCs.

Regions	Cluster size	Peak *T* value	MNI Coordinate (mm)		
			*X*	*Y*	*Z*
MFG	1773	−5.5839	±42	24	42
FG	236	−4.9282	±27	−78	−15
MSFG	131	−5.3559	±9	60	18
PG	117	−4.7597	±48	−18	42

MFG, middle frontal gyrus; FG, fusiform gyrus; MSFG, medial superior frontal gyrus; PG, precentral gyrus (voxel *p* < 0.005, cluster *p* < 0.05, GRF correction, cluster size > 64 voxels).

### 3.3. Correlations analyses

**Figure 2:**
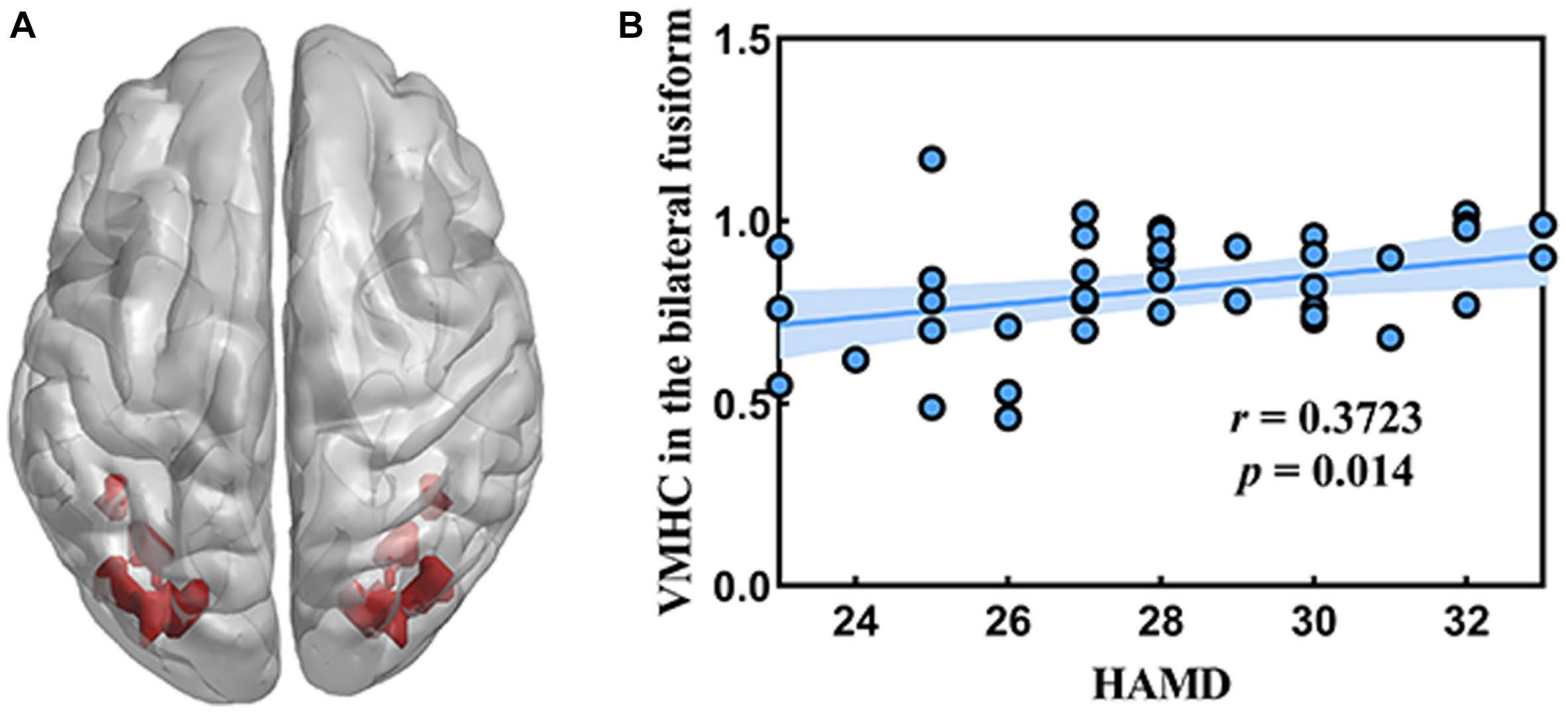
Correlation between the VMHC value in the bilateral fusiform gyrus and HAMD. **(A)** The location of bilateral fusiform gyrus in the whole brain. **(B)** The result of correlation analysis.

Figure 2 showed the correlation analysis between the VMHC and clinical characteristics. The VMHC values of different brain regions of all subjects were extracted by using RESTplus V1.25 software based on MATLAB r2017b platform. A positive correlation was observed between the VMHC value of the bilateral fusiform gyrus and HAMD (*r* = 0.3723, *p* = 0.014).

### 3.4. Support vector machine

**Figure 3:**
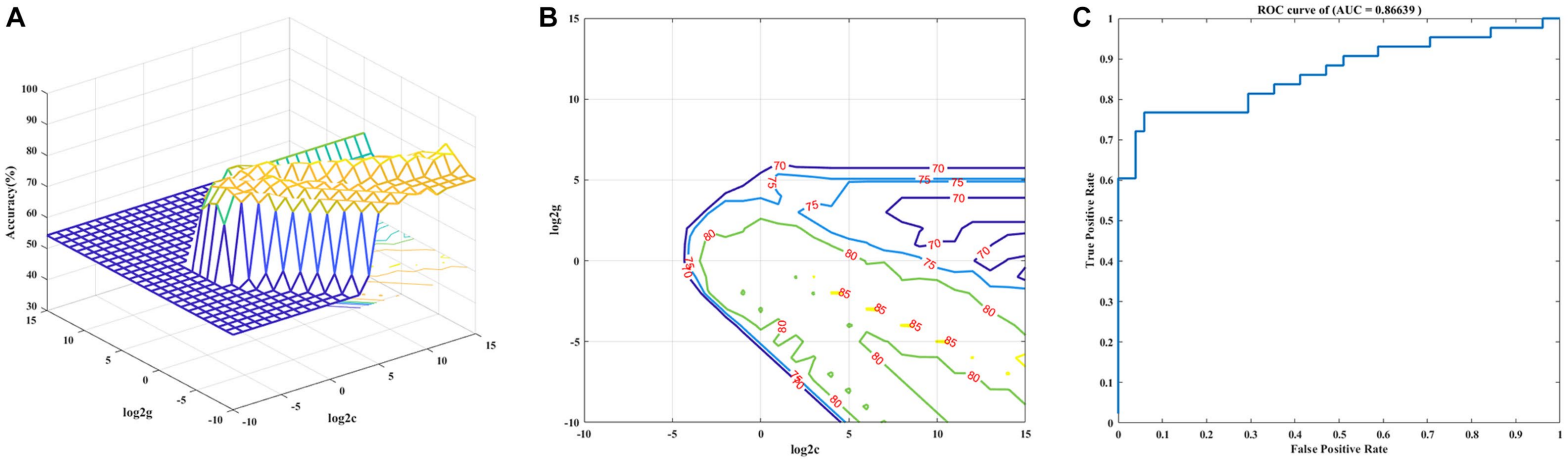
Visualization of classifications based on VMHC values through the support vector machine method. **(A)** The optimal parameters selection of SVM models by the grid search method (3D view). **(B)** The SVM parameters selection results with contour map (2D view). **(C)** Receiver operating curves assessing SVM performance.

The decreased VMHC values of these four brain regions in MDD patients were analyzed by the SVM method. The four clusters were used as features separately or together. The receiver operating curves (AUCs) of models were as follows: MFG of 0.86, FG of 0.82, MSFG of 0.79 and PG of 0.76. The decreased VMHC in the MFG showed the highest diagnostic accuracy of 81.91%, with a sensitivity of 74.42% and a specificity of 88.24%. Based on the results of the SVM, the combination of decreased VMHC in the four clusters produced the highest AUC of 0.87, with an accuracy of 86.17%, a sensitivity of 76.74%, and specificity of 94.12% together (See Table 3 and Figure 3).

**Table 3:** SVM classification performances.

Regions	Best c	Best g	Accuracy (%)	Sensitivity (%)	Specificity (%)	AUC
MFG	0.5	8	81.91	74.42	88.24	0.86
FG	512	32	80.85	76.74	84.31	0.82
MSFG	0.25	8	76.60	65.12	86.27	0.79
PG	32	512	76.60	79.07	74.51	0.76
All	32,768	0.0156	86.17	76.74	94.12	0.87

MFG, middle frontal gyrus; FG, fusiform gyrus; MSFG, medial superior frontal gyrus; PG, precentral gyrus. A combination of all clusters built the best SVM model. The best c was 32,768; The best g was 0.0156; The AUC of best model was 0.87.

## 4. Discussion

Our research compared the brain activity of MDD patients with that of HCs using the VMHC method. The results showed that compared to the HCs, the MDD group had decreased VMHC in the bilateral MFG, FG, MSFG, and PG which represented the decreased synchronization and information exchange. Additionally, a positive correlation was found between VMHC value of the bilateral FG and HAMD scores in MDD patients. Based on the SVM results, a combination of decreased VMHC value in the four clusters had relatively the highest AUC, sensitivity, specificity and accuracy.

The MSFG and the MFG are both important components of the prefrontal cortex which participate in a variety of neural functions. The MSFG is responsible for working memory, stress perception, regulation of loss aversion and behavior (41). Stress perception refers to the ability to perceive various negative external factors, which often serves as an important factor in predicting the occurrence of depression. The involvement of the MFG in emotional processing is related to psychological resilience (42, 43). The MSFG and MFG are important components of the default network and frontal parietal network. The default network plays an important role in emotional processing, self-referencing psychological activities and recalling previous experiences (44, 45). And the frontoparietal network is an important cognitive functional network that participates in controlling and regulating cognitive activities in the brain (46). The depressed patients showed substantial changes in the BOLD signal in the left MSFG relative to HCs (47) and the MSFG was demonstrated a high level of diagnostic accuracy in the late-life depression (48). Additionally, Lan et al. observed that MDD group had higher fALFF value in the right MFG (49). The MDD patients with somatic symptoms exhibited lower ReHo value in the right MFG (50) and the depressive patients had less pronounced activation of MFG in response to both positive and negative images (51). Several studies found that the abnormal interhemispheric homotopic functional connectivity in the bilateral MSFG and MFG in different types of depressive group, such as MDD with and without anhedonia, recurrent MDD and MDD with gastrointestinal symptoms (52–54). We also discovered that first-episode MDD group had lower VMHC in the MSFG and MFG compared to HCs. This indicated the importance of the homotopic connectivity between these two brain regions in the pathogenesis of depression.

The FG, known as the lateral occipitotemporal gyrus, is the cerebral cortex between the temporal lobe and the occipital lobe (55). The FG, as a crucial component of the visual recognition network, is mainly responsible for the perception and processing of emotion during face stimulus presentation. It involves in higher-order vision processing and is probably most well-known for its involvement in visual face processing, although it also plays an important role in the visual processing of body parts, objects, places and word forms (56). K and V et al. reported that MDD patients had abnormal volume in the FG related to alexithymia in comparison with healthy controls (57). And the patients with MDD have shown significantly decreased local gyrification index in the right FG and decreased functional connectivity between the right FG, right superior temporal gyrus and sensorimotor areas (precentral and postcentral gyrus) (58). Moreover, Korgaonkar et al. revealed decreased fractional anisotropy in the temporal lobe involving the FG in melancholic MDD (59). Subjects with cognitive vulnerability to depression have the increased ALFF in the left FG (60) and increased fALFF value in the FG was related to some depressive symptoms in MDD patients (61). Otherwise, one study showed that MDD group had decreased ReHo values were seen in the right FG compared with HCs (62). The MDD patients exhibited that significant decreased VMHC in the FG and a negative correlation was found between VMHC of the FG and illness duration relative to healthy controls (32, 34). Interestingly, the VMHC value of the bilateral FG was positively correlated with the HAMD in our study. This may be the transitional stage of decompensatory period. The consistency and synergy of bilateral FG was enhanced with the more severe depression, which may be related to the compensatory enhancement of information exchange and integration.

The PG was part of the central executive network. Several studies have suggested that the PG changed in patients with depression. L. Wang et al. observed that MDD patients had the significant altered ALFF and fALFF value in the precentral gyrus (63). There were significant negative correlations between the abnormal fALFF in the right precentral gyrus and the change of Beck Scale for Suicidal Ideation at baseline and between the abnormal ALFF in the right precentral gyrus and the change in HAMD. Furthermore, reduced ReHo value in the right precentral gyrus have been reported in the unipolar depression group (64). The somatic depression also exhibited that lower ReHo value in the left precentral gyrus and ReHo value in the left precentral gyrus was positively correlated with cognitive factor scores of the HAMD-17 compared to non-somatic depression (50). Additionally, Shan et al. found that the melancholic patients displayed the decreased VMHC value in the precentral gyrus and the SVM analysis results showed that the VMHC value between the bilateral precentral gyrus may serve as underlying imaging indicators to distinguish melancholic patients from non-melancholic MDD (36). The Treatment Resistant Depression group had significantly lower VMHC values in the precentral gyrus as compared to the treatment sensitive depression group (33). Our results revealed that the first-episode MDD patients exhibited aberrant VMHC value in the precentral gyrus, which was roughly consistent with the previous findings even though different types of depression. Decreased coordination was discovered throughout other brain areas, involving in bilateral insular, putamen, posterior cingulate cortex, cuneus and superior temporal gyrus (35, 65, 66). The different results may be related to the sample size, the severity and course of depression, medication or other interventions, multiple comparison correction methods and statistical threshold.

At present, clinical symptoms are mostly used for MDD diagnosis. Machine learning is an objective measurement that may might increase the accuracy of MDD diagnostic reliability. The ROC analysis was carried out to assess the effectiveness of the SVM classifier. The SVM model in our study showed good performance for MDD, with an accuracy of 86.17%, sensitivity of 76.74%, specificity of 94.12% and AUC of 0.87 based on the leave-one-out cross validation technique. As a result, aberrant VMHC signal values in these brain regions may serve as potential imaging markers for discriminating MDD patients from HC. This study had some limitations. Firstly, the sample size was small, which might reduce the statistical effectiveness and affect the stability of the results. Secondly, the study had a cross-sectional design, lacking longitudinal observation of depression. These patients can be followed up to elaborate on the pathological mechanism of the disease in the future. Thirdly, a weak correlation existed between the depression scales and the VMHC value. This may be related to the small sample size and the depression levels of the included patients. In conclusion, we found the altered VMHC of the MFG, FG, MSFG, and PG in MDD patients, indicating that the impairment of these brain areas may contribute to the pathogenesis of depression. In the future, we can combine T1 and DTI technology to further explore the neuroimaging mechanism of depression from a multimodal perspective.

## 5. Conclusion

In our study, MDD patients exhibited decreased VMHC value in the MFG, FG, MSFG and PG. The VMHC value of FG was positively correlated with the total HAMD scores. Moreover, SVM analysis results showed that a combination of the VMHC values of all clusters demonstrated the highest area under the curve (AUC), which may be a potential neuroimaging marker for the MDD. According to this study, it highlighted the importance of decreased coordination between hemispheres in these brain regions into the pathophysiology of MDD and VMHC values could also serve as a potential imaging biomarker for diagnosing MDD.

## Data availability statement

The original contributions presented in the study are included in the article/supplementary material, further inquiries can be directed to the corresponding author.

## Ethics statement

The studies involving humans were approved by this study received ethical approvals from the Ethics Committee of Guangdong Sanjiu Brain Hospital and the Ethics Committee of Southern Medical University. The studies were conducted in accordance with the local legislation and institutional requirements. The participants provided their written informed consent to participate in this study.

## Author contributions

ZL designed the study. YB, WY, SW, TX, YW, SH, CZ, SX, SK, and WK recruited the patients and collected the fMRI data. QC sorted all imaging data and carried out the analysis and wrote the manuscript. The manuscript was reviewed by ZL. All authors contributed to the article and approved the submitted version.

## Funding

This study was funded by the National Natural Science Foundation of China (Nos. 81873170, 81230085 and 82004091), administration of Traditional Chinese Medicine of Guangdong Province (No. 20201089), the National Natural Science Foundation of China (No. 82204813) and the Guangzhou Science and Technology Fund (No. 2023A04J1859).

## Conflict of interest

The authors declare that the research was conducted in the absence of any commercial or financial relationships that could be construed as a potential conflict of interest.

## Publisher’s note

All claims expressed in this article are solely those of the authors and do not necessarily represent those of their affiliated organizations, or those of the publisher, the editors and the reviewers. Any product that may be evaluated in this article, or claim that may be made by its manufacturer, is not guaranteed or endorsed by the publisher.

## Footnotes

^1^ https://www.fil.ion.ucl.ac.uk/spm/
